# Antibacterial Nanocomposites Based on Thermosetting Polymers Derived from Vegetable Oils and Metal Oxide Nanoparticles

**DOI:** 10.3390/polym11111790

**Published:** 2019-11-01

**Authors:** Ana Maria Diez-Pascual

**Affiliations:** Department of Analytical Chemistry, Physical Chemistry and Chemical Engineering, Faculty of Sciences, Alcalá University, 28871 Madrid, Spain; am.diez@uah.es; Tel.: +34-918-856-430

**Keywords:** metal oxide nanoparticles, vegetable oils, thermosetting polymers, antibacterial properties, nanocomposites, reactive oxygen species

## Abstract

Thermosetting polymers derived from vegetable oils (VOs) exhibit a wide range of outstanding properties that make them suitable for coatings, paints, adhesives, food packaging, and other industrial appliances. In addition, some of them show remarkable antimicrobial activity. Nonetheless, the antibacterial properties of these materials can be significantly improved via incorporation of very small amounts of metal oxide nanoparticles (MO-NPs) such as TiO_2_, ZnO, CuO, or Fe_3_O_4_. The antimicrobial efficiency of these NPs correlates with their structural properties like size, shape, and mainly on their concentration and degree of functionalization. Owing to their nanoscale dimensions, high specific surface area and tailorable surface chemistry, MO-NPs can discriminate bacterial cells from mammalian ones, offering long-term antibacterial action. MO-NPs provoke bacterial toxicity through generation of reactive oxygen species (ROS) that can target physical structures, metabolic paths, as well as DNA synthesis, thereby leading to cell decease. Furthermore, other modes of action—including lipid peroxidation, cell membrane lysis, redox reactions at the NP–cell interface, bacterial phagocytosis, etc.—have been reported. In this work, a brief description of current literature on the antimicrobial effect of VO-based thermosetting polymers incorporating MO-NPs is provided. Specifically, the preparation of the nanocomposites, their morphology, and antibacterial properties are comparatively discussed. A critical analysis of the current state-of-art on these nanomaterials improves our understanding to overcome antibiotic resistance and offers alternatives to struggle bacterial infections in public places.

## 1. Introduction

Over the past few decades, the manufacture and applications of synthetic polymeric materials exhibited an extraordinary boost. Nonetheless, concerns regarding diminution of fossil resources and the sharp rise in petroleum cost, health-related issues together with stringent environmental government protection policies have led to an incessantly increasing attention to the development of sustainable, safe, and environmentally friendly plastics from renewable resources. Despite the current growth of renewable polymers, their contribution to the profitable market is still very low mainly due to their expensiveness and inferior performance compared with synthetic polymers based on petroleum feedstock [1]. The key challenge for the biorenewable industry is to produce materials with properties that match or even go beyond those of petroleum-based ones. The most widely used renewable resources are plant oils, polysaccharides, and proteins [2].

### 1.1. Thermosetting Polymers Obtained from Vegetable Oils

**Scheme 1:**
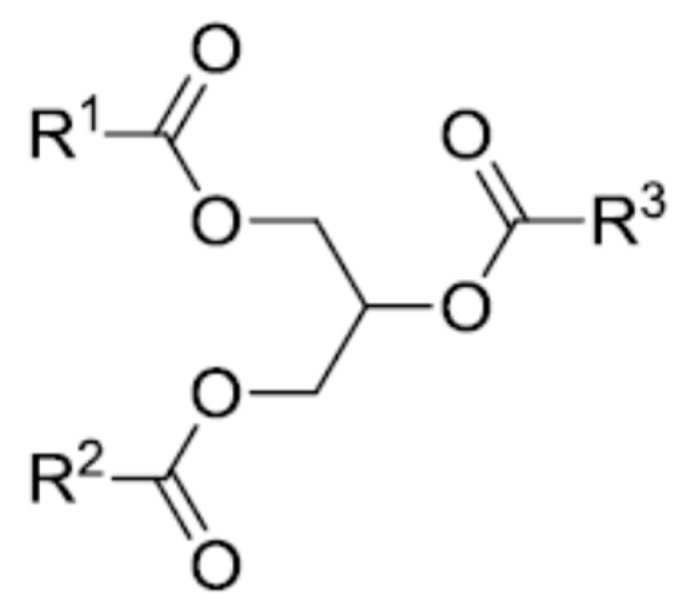
General structure of a triglyceride from a plant oil (R^1^, R^2^, and R^3^ represent fatty acid chains).

Plant or vegetable oils (VOs) can be used as raw materials for polymers due to their abundance in nature, moderate cost, biodegradability, and very low toxicity [3]. Furthermore, their polar character bestows improved antibacterial properties to the resulting polymeric systems. The main elements of VOs are triglycerides, which are the result of the esterification of glycerol with three fatty acids (Scheme 1). Fatty acids represent 95% of the whole weight of triglycerides and their composition changes depending on the plant, harvest, season, growing conditions, and purification methods [4]. The fatty acids have different levels of saturation and unsaturation [5], being the unsaturated part the one that can be functionalized to yield the liquid resin to be used for the polymer synthesis. Many reactive points of the triglycerides—like double bonds, allylic carbons, and ester groups—can be used to introduce polymerizable groups. The chain length of fatty acids in triglycerides occurring in nature typically varies between 16 and 20 carbon atoms. Table 1 collects the most frequent fatty acids present in vegetable oils. Most of them exhibit a straight chain with an even number of carbons and double bonds in a cis configuration.

Table 2 summarizes the proportions of the different fatty acids in the most frequent VOs. The chemical and physical properties of VOs are strongly influenced by the level of unsaturation, which can be calculated by measuring the iodine value (IV), which indicates the amount of iodine (mg) that reacts with the C=C double bonds in 100 g of the VOs; the higher the IV value, the more number of C=C per triglyceride of the VOs. Thus, VOs can be divided into drying oils (IV > 130), semi-drying oils (100 < IV < 130), and non-drying oils (IV < 100). The IV values of the most widespread VOs are also collected in Table 2.

Linseed, soybean, sunflower, palm, olive, cottonseed, castor, and rapseed oils are the most frequently used for the synthesis of biopolymers [6,7], and can be used in a number of applications including lubricants, coatings, soaps, cosmetic products, biodiesel, and so forth. Different derivatives of VOs have been used as polymerizable monomers in radiation-curable systems to yield flexible but tough resins such as epoxy, urethane, and polyester [8]. Combinations of epoxidized soybean oil and traditional epoxy resins or their copolymers with styrene and divinylbenzene as well as polyurethane resins derived from castor oil have been used to fabricate composite materials with outstanding mechanical and barrier performance [9]. Furthermore, epoxidized vegetable oils (EVOs) such as soybean and palm have been used in UV-curable coating systems [7,8]. Despite the use of VOs in paints and coatings is quite old, emphasis is currently being made on these materials to attain novel properties, superior performance together with environmentally friendliness at reasonable costs [10].

Different VOs have been used for the preparation of cationic polyurethane (PU) coatings with antimicrobial activity to be applied in the biomedical or food packaging industries. For instance, Xia et al. [11] developed PU coatings based on five amino polyols derived from soybean oil and studied the influence of their structure and functionalities on the antimicrobial, mechanical, and thermal properties. Those comprising N-methyldiethanolamine (MDEA)- and N-ethyldiethanolamine (EDEA), with the shortest side chains, exhibited the best antibacterial action. The same authors [12] modified the crosslinking density of the materials using soybean polyols with different number of hydroxyl moieties as starting materials. Reduction of the crosslink density by lessening the hydroxyl number of the soybean polyol promoted the physical interaction with the target bacteria via increasing the molecular chain mobility, thus resulted in superior antibacterial properties. In another study, Liang et al. [13] employed castor oiland MDEA to prepared antimicrobial PUs, and improved action was also observed via reduction of the polyol functionality.

On the other hand, polymers derived from VOs display reduced mechanical and barrier properties, which restrict their realistic applications [1]. Thus, their brittleness, elevated gas and vapor permeability, and low heat distortion temperature have limited their commercial uses. To solve these issues, different strategies have been reported, including mixing with other polymers [14] or incorporating nanofillers [15,16,17,18]. The addition of nanofillers to thermosetting polymers based on VOs results in bionanocomposites with enhanced performance [15,16]. Bio-based PUs show poor antimicrobial activity, which can be significantly improved via incorporation of regularly dispersed metal or metal-oxide nanoparticles [19].

### 1.2. Antimicrobial Effect of Metal-Oxide Nanoparticles

Metallic elements combine with oxygen to form metal oxides (MOs), which have been demonstrated to interact with bacteria by means of electrostatic interactions that modify the prokaryotic cell wall and damage the DNA via reactive oxygen species (ROS) generation [20]. MOs have attracted a lot of attention as a nanomaterial that can be synthesized into a specific size and shape. Bearing these in mind, metal oxide nanoparticles (MO-NPs), in particular those based on ZnO, TiO_2_, CuO, and MgO, are being used as antimicrobial agents [20]. The mechanism of MO-NPs as antibacterial agents is influenced not only by the chemical composition, but also by the shape, size, solubility, degree of agglomeration, and surface charge of the NPs [21]. In particular, the morphology and size of NPs has been demonstrated to be crucial in the antimicrobial efficacy: the smallest spherical NPs were the most effective in destroying the bacteria, since are more prone to go through the bacterial cell walls due to their increased surface area to volume ratio [22]. On the other hand, low NP solubility has been demonstrated to bring out a reduced cytotoxic response [22]. The NP solubility also influences its tendency to agglomerate, which controls the NP–cell interactions. Thus, highly agglomerated NPs are not able to enter a cell or generate a considerable amount of ROS [23]. This can be due to the decrease in the surface area. Nevertheless, when NPs are well dispersed, they increase the interactions with the cells and the ROS fabrication. In addition, the NP surface charge influences the bactericide action. NPs with positive zeta potentials allow for electrostatic interactions with bacteria comprising negatively charged surfaces. This promotes NP penetration into the cell membrane. High zeta potentials support a strong interaction, provoking membrane disruption, bacteria flocculation, and viability lessening. From all the above facts, it can be concluded that the antimicrobial effects of MO-NPs can be tailored by controlling their surface charge, size, and surface morphology. 

#### 1.2.1. Antimicrobial Effect of Zinc Oxide

Zinc is a fundamental nutrient that plays a key role in growth of mammals. At the nanoscale, ZnO nanostructures have involved a lot of interest within the scientific community due to their inexpensiveness, ease of use, possibility of functionalization and biocompatibility. This nanomaterial is a semiconductor with a wide bandgap (3.37 eV), hence widely used for UV-Vis optical devices [24]. Furthermore, its bandgap can be modified by mixing with MgO and CdO, which is interesting for optoelectronic applications. It possesses large specific surface area, radiation resistance, good mechanical properties [25] (i.e., a Young’s modulus close to 100 GPa and hardness of about 5 GPa), low coefficient of thermal expansion and high thermal conductivity (around 1 W cm^−1^ K^−1^) [26]. At ambient temperature and pressure, ZnO crystallizes in the wurtzite structure. It is a hexagonal lattice including two interconnecting sublattices of Zn^2+^ and O^2−^, in which each Zn^2+^ is surrounded by a tetrahedra of O^2−^ and vice-versa [24]. This structure results in polar symmetry along the hexagonal axis, being accountable for the piezoelectricity and spontaneous polarization that has been observed in ZnO.

**Figure 1:**
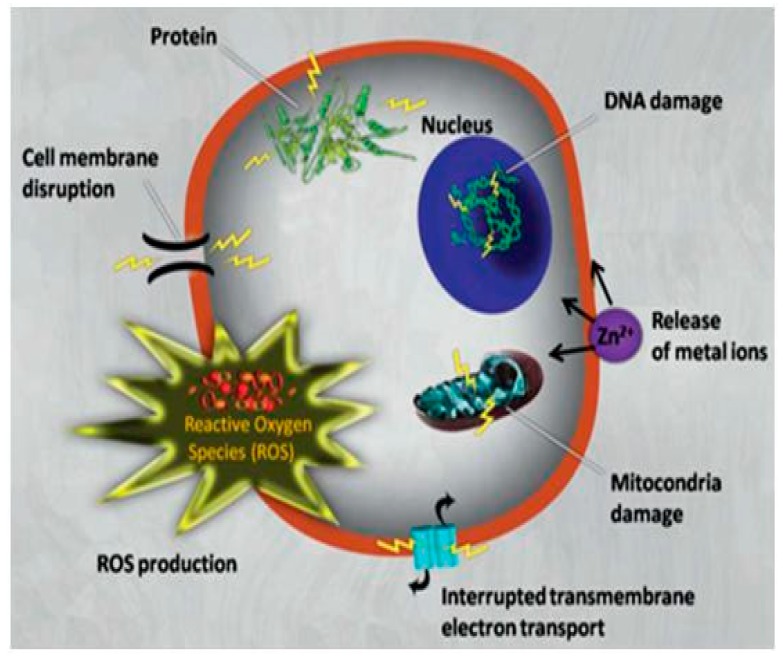
Mechanisms of antimicrobial activity of ZnO nanoparticles. Adapted from [27], copyright 2012, with permission from Elsevier.

The main benefits of using these MO-NPs as antimicrobial agents are that they enclose mineral elements vital for humans and show strong action when provided in small quantities. The antimicrobial action of ZnO NPs has been endorsed to different mechanisms [27,28], as schematized in Figure 1: a) bringing on oxidative stress due to formation of reactive oxygen species (ROS). Upon light exposure, electron–hole pairs are formed on the ZnO surface; the holes split water molecules and cause ROS formation, explicitly •OH, H_2_O_2_, and •O^2−^, which can subsequently interact with proteins, DNA, and lipids of the bacteria, thereby inducing cell death; b) cell membrane disruption due to accumulation of the NPs at the bacterial membrane followed by NP internalization within the cell and destruction of organelles; c) release of Zn^2+^ ions that join to the bacteria membrane; d) lipid peroxidation can occur on the bacterial membrane, weakening membrane integrity, and promoting cell lysis. 

The attachment of the ZnO-NPs to the bacterial membrane is a crucial step in the three aforementioned mechanisms. The way the NPs anchor to the membrane is not fully clear yet, though it seems that arises from electrostatic forces. Subsequent to the attachment, “pitting” takes place at the membrane due to ROS generation, which lethally harms the cells [29].

ROS generation has been reported to increase with increasing ZnO surface area while decreasing level of crystallinity and particle size [30]. Even though ZnO has been used as an bactericide agent against both Gram-positive (*B. subtilis*, *S. aureus*) and Gram-negative bacteria (*P. aeruginosa*, *E. coli*), it displays higher activity towards Gram-positive ones, ascribed to different reasons, the most important being the difference in the membrane thickness [17,31]. Gram-positive bacteria contain a cell wall composed of peptidoglycans, teichoic, and lipoteichoic acids that it is simpler to be penetrated in comparison to the complex wall of Gram-negative ones, with an outer membrane of lipopolysaccharides and a peptidoglycan layer.

#### 1.2.2. Antimicrobial Effect of Titanium Oxide

TiO_2_ nanostructures are n-type semiconductors with exceptional properties for optics, photonics and electronic applications [32]. They are non-toxic, low reactive, rather inexpensive and environmentally friendly, and possess outstanding mechanical properties, low coefficient of thermal expansion, high thermal conductivity, and strong UV absorption.

In nature, three main polymorphs of TiO_2_ can be found: anatase (tetragonal), brookite (orthorhombic), and rutile (tetragonal), the former being the most commonly employed in photocatalytic applications [33]. Furthermore, it is the most toxic form, thus used to obtain antimicrobial properties [34], being employed in medical devices, household cleaning products, air-conditioning surfaces, water treatment facilities, and so forth. Anatase is the least thermodynamically stable polymorph as a bulk phase, although it generally predominates when the grain size is below 100 nm. Nanostructured anatase-TiO_2_ can be synthesized by a large number of techniques including liquid-phase sol–gel, microemulsion, hydro- and solvothermal, aerogel, sonochemical or surfactant-templated methods [35]. 

TiO_2_ NPs have been used as bactericide agents for both Gram-positive and Gram-negative bacteria [35], and their properties are photodependent. Similarly to ZnO, they can produce different types of ROS, including hydroxyl radicals •OH, superoxide radicals •O^2−^ and singlet oxygen (^1^O_2_) [36], as well as other species like H_2_O_2_ or O_2_ that can disrupt the bacteria cell membrane. In particular, hydroxyl radicals seem to play the most important role on the antibacterial action of these NPs. Due to peroxidation, these free radicals affect the lipopolysaccharide, peptidoglycan, and phospholipid bilayers [37]. Owed to their photo-dependent properties and large effective surface area, TiO_2_ NPs are frequently applied in surface coatings. Furthermore, the smaller the NPs, the more prone to enter the bacteria cell due to the increased surface area to volume ratio. In contrast, highly agglomerated NPs are not able to penetrate the cells or make a significant amount of ROS. Agglomeration is influenced by a number of factors, including the hydrophobicity of the material, the interactions with the dispersed medium (i.e., pH, protein content), and the surface charge [38].

#### 1.2.3. Antimicrobial Effect of Copper Oxide

CuO is a p-type semiconductor with a narrow band gap of 1.2–1.9 eV that has attracted special attention due to its valuable physical properties including high temperature superconductivity, electron correlation effects, and spin dynamics [39]. It is quite economical, easily mixed with polar liquids (i.e., water) and polymers, and stable in terms of both chemical and physical properties. The crystal structure of CuO belongs to the monoclinic space group C2/c; each Cu^2+^ is bonded in a square co-planar geometry to four equivalent O^2-^ [40]. Elemental copper and its oxides have been renowned as antimicrobial agents by the US Environmental Protection agency (EPA). This makes them suitable for numerous applications in paints, fabrics, agriculture, and in hospitals both as powder and as coating. 

CuO antibacterial action has been ascribed to a rapid lessening in cell membrane integrity and production of ROS, namely H_2_O_2_, hydroxyl radicals •OH, superoxide radicals •O^2−^ and singlet oxygen (^1^O_2_), being the hydroxyl radical the most common. Thus, CuO-NPs generate ROS through several mechanisms, likely including Fenton-like and Haber–Weiss reactions at the nanoparticle surface or in solution via release of Cu^2+^ dissolved from the nanoparticle surface [41]. Other proposed mechanisms include DNA damaging via formation of a DNA/Cu^2+^/H_2_O_2_ complex or Cu^2+^-bound•OH as the damaging species [42]. ^1^O_2_ can also be formed in the presence of CuO-NPs under oxidative stress conditions, and it decomposes into •OH. Furthermore, in bacterial cells, Cu^2+^ ions are reduced by sulphydryl to Cu^+^, and the reduced ions are responsible for causing oxidative stress via Cu^+^-driven ROS. Nonetheless, the exact pathway of antimicrobial action of CuO is not clear in the literature [40].

#### 1.2.4. Antimicrobial Effect of Iron Oxide

Fe_3_O_4_ occurs in nature as the mineral magnetite. It is a ferrimagnetic oxide, with a Curie temperature of 858 K, with semiconducting properties, with conductivities ranging from 10^2^–10^3^ Ω^−1^cm [43]. Fe_3_O_4_ nanomaterials have attracted incredible interest since they are biocompatible and show exceptional electric, thermal, mechanical, and magnetic properties. Nanostructures with a variety of morphologies have been synthesized and applied in fields like lithium-ion batteries, wastewater treatment, magnetic resonance imaging contrast agents, therapeutics for cancer treatment, and radiation oncology. The crystal structure of magnetite corresponds to the cubic inverse spinel pattern in which the oxygen ions form a cubic face centered packing, and the iron ions locate at interstitial tetrahedral and octahedral sites [44].

The antimicrobial activity of magnetite NPs is believed to be the result of their interaction with bacterial membranes and their penetration into the bacterial cell, causing membrane damage and inactivation of bacteria. H_2_O_2_, which is produced from the metabolic activity of the bacteria, can react with the species present in magnetite: different oxido-reduction reactions take place involving both Fe^3+^ and Fe^2+^, (Fenton-like and Haber–Weiss reactions), leading to the formation of ROS, in particular •OH and •HO_2_ free radicals. The ROS produced at the microorganism surface depolarizes the bacterial membranes, causing oxidative stress and cell membrane damage [45].

## 2. Preparation of Vegetable Oil-Based Thermosetting Polymers Incorporating Metal Oxide Nanoparticles

### 2.1. Synthesis of Acrylated Epoxidized Linseed Oil (AELO)/TiO_2_ Nanocomposites

Firstly, the epoxidized linseed oil (ELO) was synthesized from the vegetable oil in a four-necked glass flask incorporating a mechanical stirrer, a thermometer, and a reflux condenser. The VO and formic acid were mixed and subsequently H_2_O_2_ was added dropwise. Then, the reaction continued for 4 h; when the product was cooled down, it was filtered and washed repetitively with distilled water, and finally dried in an oven overnight. 

**Scheme 2:**
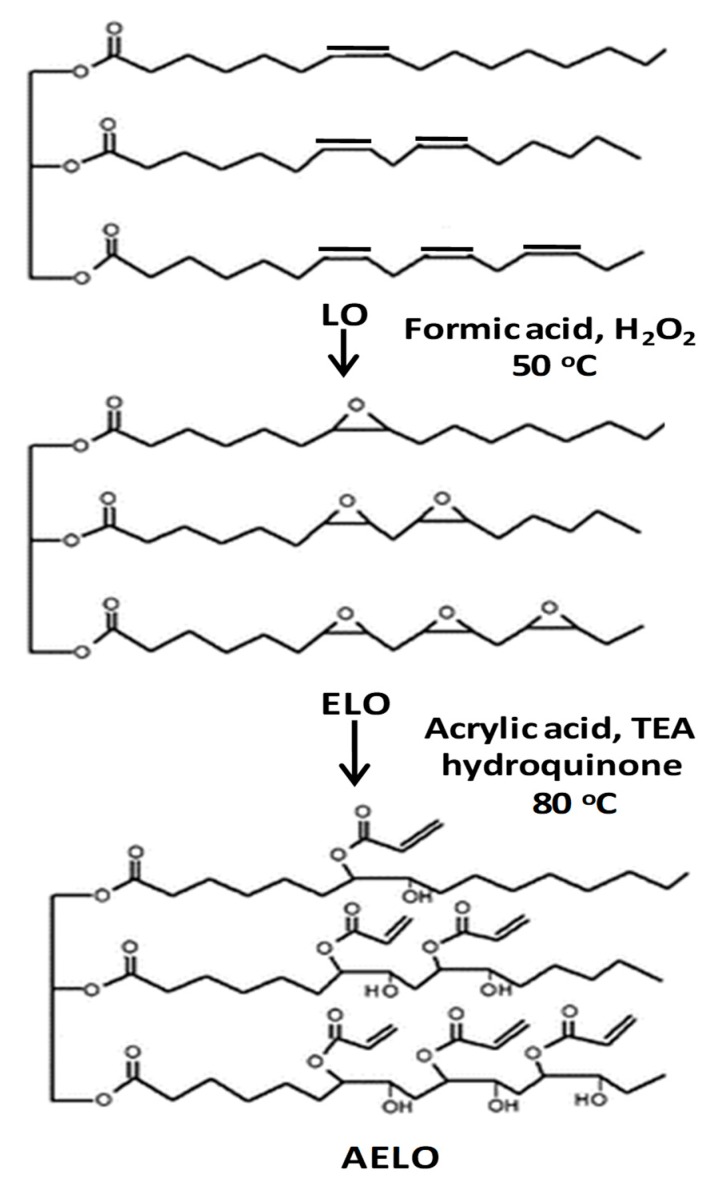
Representation of the synthesis of AELO resin. Reprinted from [46], copyright 2015, with permission from the Royal Society of Chemistry.

Acrylated epoxidized linseed oil (AELO) was prepared from ELO using acrylic acid as ring opening agent, triethylamine (TEA) as a catalyst and hydroquinone as a free radical inhibitor. In short, a mixture of ELO and acrylic acid was heated in the presence of TEA for about one day until an acid value of ~8 mg KOH/g was attained. Unreacted acrylic acid and TEA were eliminated by extraction. The esterification extent was determined as 88% measuring the acid values at the start and finish of the reaction. The schematic representation of the synthesis of AELO is presented in Scheme 2 [46]. 

AELO resin was then crosslinked with trimethylolpropane trimethacrylate monomer using benzophenone as photoinitiator, 2-dimethylaminoethanolas activator, and TiO_2_ nanoparticles as fillers, and subjected to UV irradiation to obtain the cured nanocomposites, with nanoparticle loadings of 1.0, 2.5, 5.0, and 7.5 wt %.

### 2.2. Synthesis of Crosslinked Castor Oil (CO)/Chitosan-Modified ZnO Nanoparticles (CS-ZnO NPs)

Firstly, CS-modified ZnO NPs were synthesized by dissolving a small amount of ZnO nanopowder in an acetic acid solution. Then, the same amount of CS was added and the mixture was ultrasonicated, followed by drop-wise addition of NaOH until a pH of 10 was reached. The resulting product was heated, filtered, washed with distilled water, and dried in an oven.

**Scheme 3:**
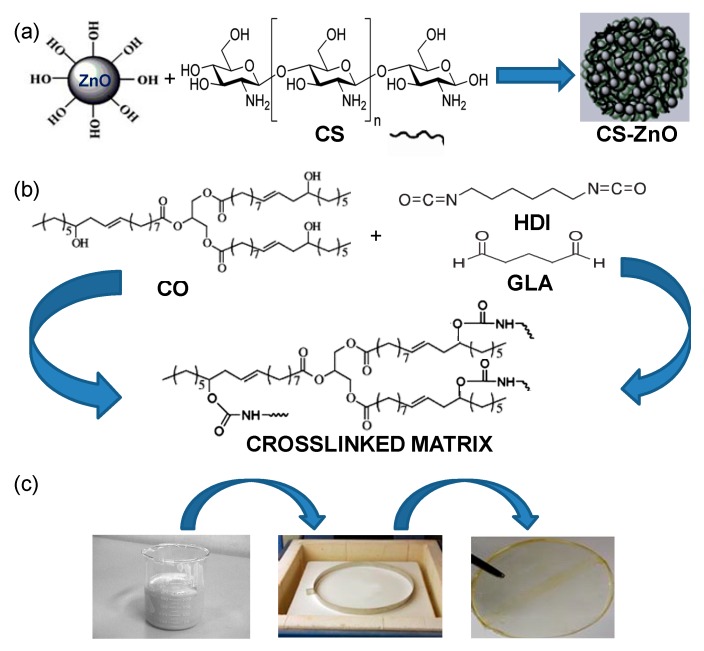
Representation of the synthesis of CO/CS-ZnO NPs nanocomposites. (**a**) CS-ZnO NPs; (**b**) CO, HDI, GLA, and the crosslinked matrix; (**c**) illustration of the film casting process. Reprinted from [18], copyright 2015, with permission from the American Chemical Society.

The nanocomposites were then fabricated by solution mixing and casting process. Briefly, CO and the desired amounts of CS-ZnO NPs to obtain loadings of 1.0, 2.5, 5.0, and 7.5 wt %, were stirred, and then hexamethylene diisocyanate (HDI) and glutaraldehyde (GLA) were added as crosslinking agents to react with the CO in the presence of stannous octoate as catalyst. The mixture was heated and then poured onto Teflon plates. Curing was performed under vacuum in an oven. The schematic representation of the synthesis of CO/CS-ZnO NPs nanocomposites is shown in Scheme 3 [18]. 

### 2.3. Synthesis of Epoxidized Soybean Oil (ESO)/ZnO Nanocomposites

Initially, the VO and formic acid were mixed in a four-necked vessel, and H_2_O_2_ was added dropwise to start the epoxidation. The reaction continued for a few hours, and then the product, the epoxidized soybean oil (ESO) was cooled down, washed and dried overnight in an oven. ESO was mixed under stirring with different amounts of ZnO nanoparticles to yield nanocomposites containing 1.0, 3.0, 5.0, and 7.0 wt % loading in the presence of 4-dimethylaminopyridine as catalyst. The nanocomposites were degassed, decanted into silicone moulds, and cured in a vacuum oven [17].

### 2.4. Synthesis of Geranium-Derived Oil (Ge)/ZnO Nanocomposites

Nanocomposites were synthesized via plasma polymerization under vacuum in a cylindrical glass tube equipped with two external Cu rings that acted as electrodes connected to a generator. The glow discharge was created by a radio frequency generator. Firstly, the pressure was reduced and subsequently the monomer gas was brought into the tube until the pressure attained a stable value. The tube had an external ceramic fiber heater, where zinc acetylacetonate hydrate powder, the Zn precursor, was placed in a ceramic boat and heated. The vapor of the precursor entered the manufacture zone, ZnO nanoparticles were formed in the gas phase and then embedded into the geranium oil (Ge) matrix during the in situ polymerization. Two nanocomposites were prepared, with input radio frequency powers of 10 W and 50 W, which resulted in NP loadings of 0.79% and 1.57%, respectively [47].

### 2.5. Synthesis of Sunflower Oil Derived Hyperbranched Polyurethane (HBPU)/Fe_3_O_4_ Nanocomposites

The sunflower oil derived hyperbranched polyurethane (HBPU) was prepared in a two step process [48]: firstly, toluene diisocyanate, butanediol and polycaprolactone were mixed under an inert atmosphere. Secondly, pentaerythritol (a branching tetra-functional) was added with the monoglyceride of sunflower oil, and the reaction continued for a few hours. Separately, Fe_3_O_4_ NPs were prepared by a coprecipitation method: FeCl_2_ and FeCl_3_ were stirred in water under a nitrogen atmosphere followed by addition of NH_4_ solution until a basic pH was attained.

Nanocomposites with different amounts of Fe_3_O_4_ (0, 5, 10, and 15 wt %) were prepared by solution casting. The NPs were dispersed in *N*,*N*-dimethyl acetamide by ultrasonication and added to the VO-derived polyurethane in the second stage of the polymerization reaction. The mixtures were stirred and then cooled down, followed by casting on inert substrates and curing.

**Scheme 4:**
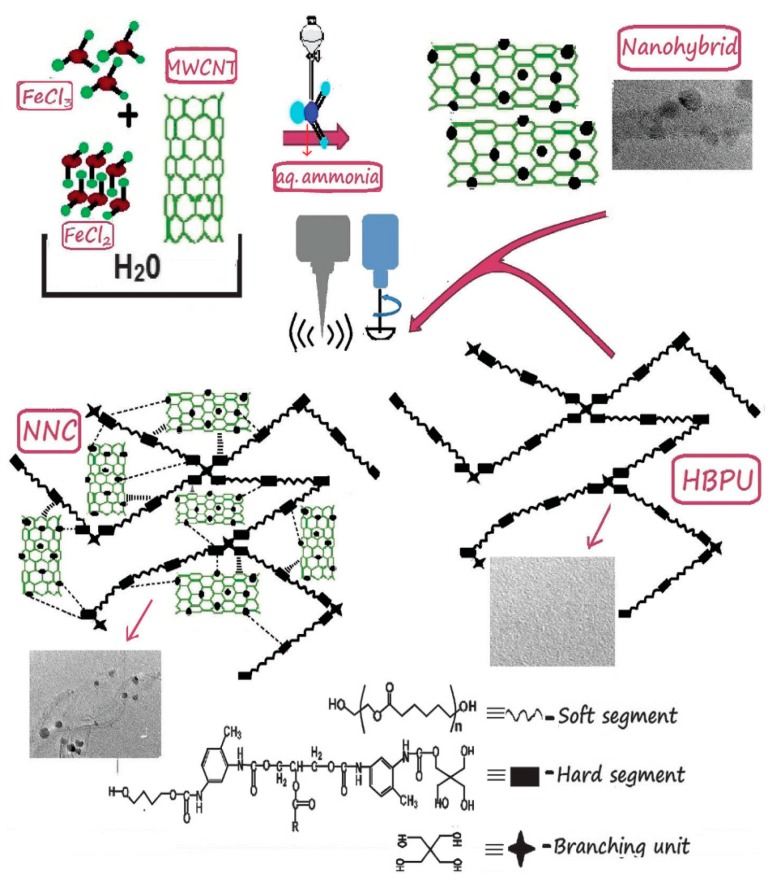
Representation of preparation of Fe_3_O_4_–MWCNT hybrids and the corresponding sunflower oil derived polyurethane nanocomposites. Reprinted from [49], copyright 2014, with permission from the Royal Society of Chemistry.

A very similar approach was used to prepared nanocomposites based on the same matrix filled with Fe_3_O_4_ nanoparticles coated with multiwalled carbon nanotubes (MWCNTs), which were prepared in a single stage reaction (Scheme 4) [49].

Initially, FeCl_2•_4H_2_O and FeCl_3_ were dispersed in water under an inert atmosphere. Separately, the required amount of MWCNTs was also dispersed in water followed by ultrasonication. The dispersed MWCNTs were then added to the first dispersion and the mixture was stirred again. Finally, aqueous NH_4_ solution was added dropwise to the mixture until a basic pH was attained.

### 2.6. Synthesis of Linseed Oil (LO) Derived Polyol/CuO Nanocomposites

The linseed oil (LO) polyol was synthesized by mixing the LO, acetic acid, and H_2_O_2_ under mechanical stirring. Then, the temperature was raised for a few hours, during which epoxidation, and hydration reactions took place. The resulting polyol was successively washed with sodium bicarbonate aqueous solution, distilled water, and sodium chloride aqueous solution, and finally dried. The nanocomposites were prepared in situ via a ‘solventless one-pot’ chemical reaction. The polyol was then mixed with phthalic anhydride under stirring, followed by addition of different amounts of copper acetate. The reaction mixture was heated and refluxed. Three nanocomposites with CuO contents of 0.04, 0.05, and 0.06 mol were prepared [50].

## 3. Morphology of Vegetable Oil-Based Thermoset Polymers with Metal Oxide Nanoparticles

The morphology of the nanocomposites plays a key role in determining their antibacterial activity, as will be discussed in a following section. Hence, it is interesting to analyze the size, shape, etc., of the developed nanocomposites.

### 3.1. Linseed Oil-Based Nanocomposites

**Figure 2:**
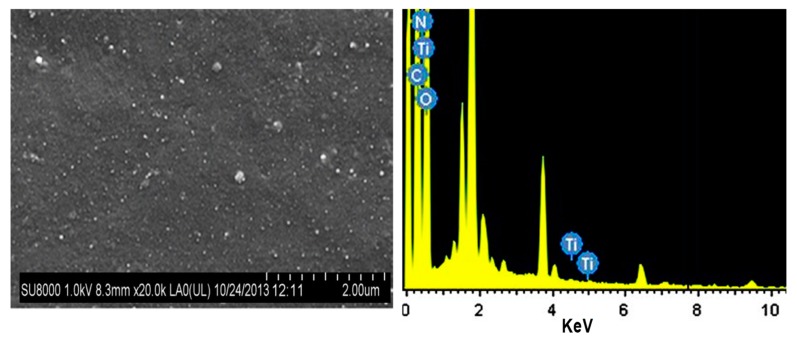
SEM micrograph (left) and EDX (right) of AELO/TiO_2_ (7.5 wt %) nanocomposite. Reprinted from [46], copyright 2015, with permission from the Royal Society of Chemistry.

The surface of AELO/TiO_2_ nanocomposites was investigated by scanning electron microscopy (SEM), and a typical illustration of the sample with 7.5 wt % loading is shown in Figure 2. The image shows a great number of spherical nanoparticles (bright spots) with an average diameter of 40 nm arbitrarily dispersed within the epoxidized VO, and also a few small clusters comprising several NPs. The interactions between the OH groups on the TiO_2_ surface and the polar groups of the AELO avoid NP agglomeration, and no particle surface treatments or interfacial modifiers are required for the manufacturing of these biomaterials, making them cheaper and more environmentally friendly. Figure 2 also shows the energy dispersive X-ray (EDX) spectrum of the composite, which corroborates that the sample contains Ti, C, N, and O atoms.

**Figure 3:**
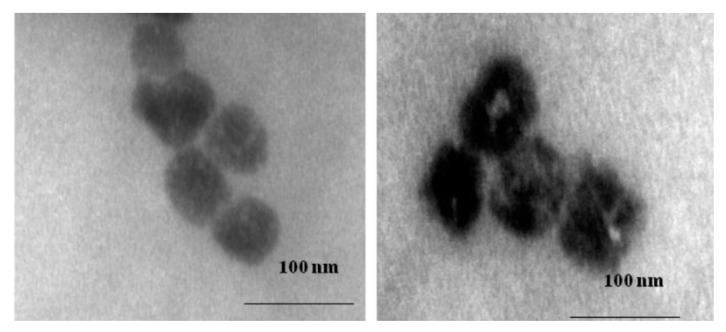
TEM micrographs of an epoxidized LO/CuO nanocomposite. Reprinted from [50], copyright 2013, with permission from Elsevier.

Transmission electron microscopy (TEM) micrographs of LO derived polyol/CuO nanocomposites reveal the presence of spherical nanosized NPs with diameters ranging between 50 and 60 nm Figure 3. With increasing NP loading, the nanocomposites become denser, hence with higher refractive index and viscosity.

### 3.2. Castor Oil-Based Nanocomposites

**Figure 4:**
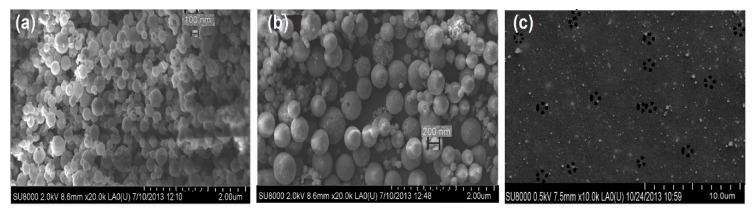
SEM images of: (**a**) as synthesized ZnO nanoparticles; (**b**) chitosan-modified ZnO nanoparticles; (**c**) cured castor oil/chitosan-modified ZnO nanocomposite (7.5 wt %). Reprinted from [18], copyright 2015, with permission from the American Chemical Society.

SEM images of ZnO NPs (Figure 4a) revealed their quasi-spherical shape, with diameters ranging from 40 to 180 nm. Furthermore, due to the big surface area and high surface energy of the NPs, some agglomeration took place, and many small clusters can be observed. Upon functionalization with chitosan, their surface turned out to be more porous, and their average size raised to 180 nm (Figure 4b). Additionally, the agglomeration decreased, since the interaction between chitosan and the NPs reduced the attractive forces between nanoparticles [18].

On the other hand, the nanocomposites were transparent and flexible, as shown in Figure 4c. The CS-ZnO NPs can be observed as bright spheres homogeneously and randomly dispersed within the CO matrix. The H-bonding interactions between the urethane moieties of the cured CO and the OH moieties of the CS-NPs avoid aggregation and improve the matrix-NP compatibility. The porosity of the nanocomposites was found to increase with increasing NP loading, from 10% for neat CO to almost 100% at 7.5 wt % NP content. This augment in porosity is valuable for wound healing applications, since would enable the absorption of higher volumes of wound exudates and promote the distribution of nutrients to the cells.

### 3.3. Soybean Oil-Based Nanocomposites

**Figure 5:**
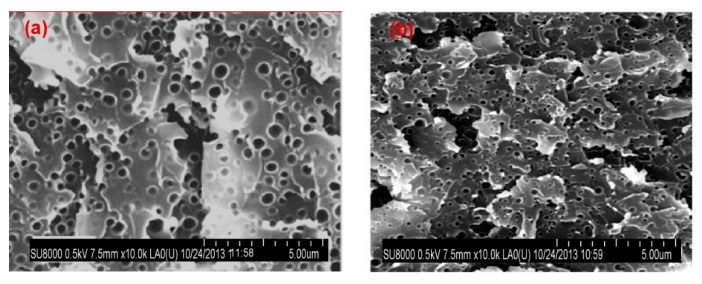
SEM images of ESO (**a**) and ESO/ZnO (5.0 wt %) nanocomposite (**b**). Reprinted from [17], copyright 2014, with permission from the American Chemical Society.

The epoxidized SO showed a sponge-like morphology (Figure 5a), with many round pores of about 300 nm size [17]. However, in the nanocomposites filled with ZnO NPs (Figure 5b), the pore dimension was about half that of the matrix, while the surface roughness increased. The spherical NPs, with an average size of 80 nm, which increased slightly with increasing loading, become visible as light spots in the images, and are very well distributed. The interactions between the polar groups of the SO and the OH moieties of the ZnO NPs avert aggregation without the necessity of particle surface treatments or compatibilizers.

### 3.4. Geranium Oil-Based Nanocomposites

SEM images were attained to assess the size and morphology of the synthesized ZnO NPs in the polymer matrix [47]. Ball-like NPs randomly and homogenously distributed were found in all the composites. Nonetheless, the NP size moderately increased with increasing power deposition, from about 60 nm at 10 W to around 80 nm at 50 W, indicating that a higher amount of NPs can be incorporated into the polymer matrix at higher deposition powers.

**Figure 6:**
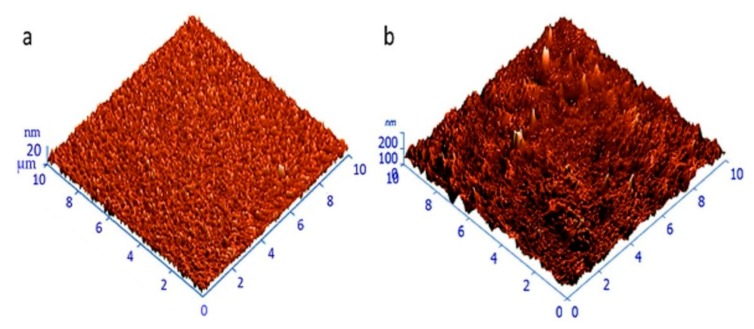
AFM images of neat Ge (**a**) and the corresponding nanocomposite with 1.57% ZnO (Zn/Ge) (**b**). Reprinted from [47], copyright 2019, with permission from Springer Nature.

The surface morphology of Ge and the nanocomposites was also examined via atomic force microscopy (AFM). Due to the soft Ge surface, the tapping mode was used. The surface of neat Ge was flat, soft, and uniform (Figure 6a), with a mean particle roughness of 0.25 nm. The average roughness increased with the incorporation of ZnO (Figure 6b), and the nanocomposites with NP loadings of 0.79% and 1.57%, which correspond to frequency powers during the plasma polymerization of 10 and 50 W, showed average roughnesses of 33.7 and 37.2 nm, respectively. The nanocomposites have a more porous surface with a random distribution of the NPs, which are present in the form of protusions.

### 3.5. Sunflower Oil-Based Nanocomposites

**Figure 7:**
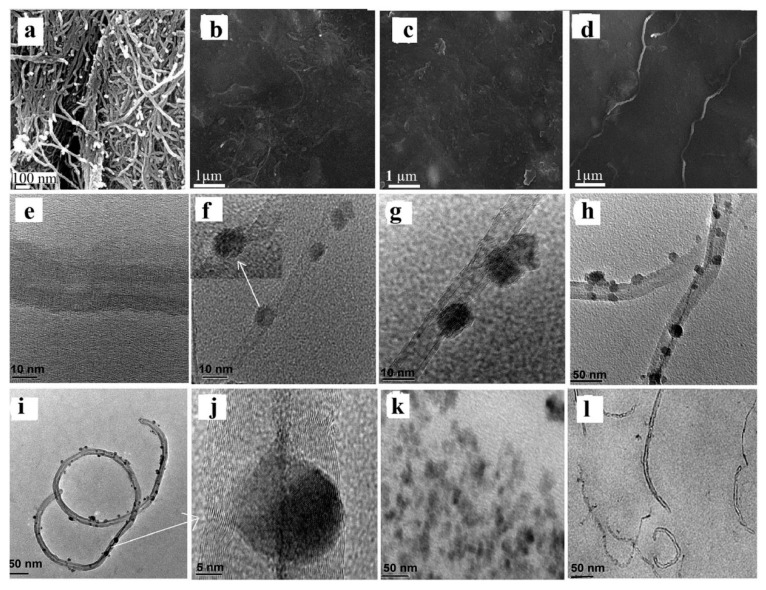
SEM images of Fe_3_O_4_–MWCNT nanohybrids (**a**), NNC (**b**), FNC (**c**), and MNC (**d**); TEM images of MWCNT (**e**), Fe_3_O_4_–MWCNT nanohybrid (**f** and **g**), NNC (**h**–**j**); FNC (**k**) and MNC (**l**). Nomenclature: carboxyl functionalized MWCNT-based nanocomposites (MNC); Fe_3_O_4_-based nanocomposites (FNC) and Fe_3_O_4_–MWCNT-based nanocomposites (NNC). Reprinted from [49], copyright 2014, with permission from the Royal Society of Chemistry.

According to SEM and TEM images, a very homogeneous dispersion of the Fe_3_O_4_ NPs and the Fe_3_O_4_–MWCNT hybrids within the VO matrix was achieved during the in situ polymerization. The MWCNTs were relatively small and short (~10 nm diameter, length < 5 μm, Figure 7d,e). Fe_3_O_4_ NPs with diameters ranging from 9 to 12 nm decorated the wall of the MWCNTs (Figure 7f,g), which was attained via debundling of the MWCNTs upon sonication and mechanical shearing. Besides, the presence of dangling bonds on the MWCNT aid the NP nucleation on the nanotubes surface (Figure 7b,h,j). The П–П interactions between the aromatic rings of toluene diisocyanate and those of the MWCNTs and the interaction between the –C=O and –N–H of the polyol resin with the OH groups of the NPs led to strong interfacial adhesion between them, Figure 7c,d [49].

## 4. Antimicrobial Activity of Vegetable Oil-Based Thermoset Polymers with Metal Oxide Nanoparticles

### 4.1. Antimicrobial Effect of Zinc Oxide-Reinforced Nanocomposites

**Figure 8:**
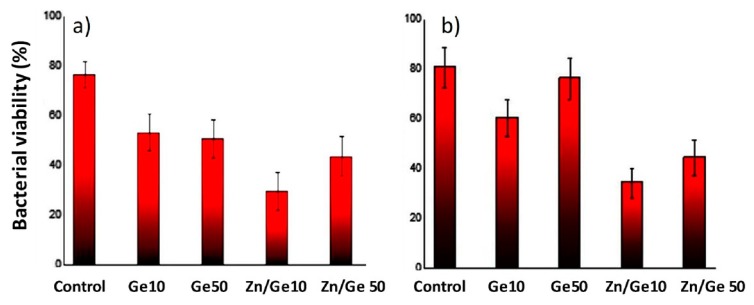
*S. aureus* (**a**) and *E. coli* (**b**) viability against neat geranium oil (Ge) and geranium oil/ZnO nanocomposites (Zn/Ge) prepared via plasma polymerization with radio frequency powers of 10 W (Ge 10 and Zn/Ge 10) and 50 W (Ge 50 and Zn/Ge 50). Adapted from [47], copyright 2019, with permission from Springer Nature.

The antibacterial activity of neat Ge and the corresponding nanocomposites with ZnO nanoparticles was investigated against two well-known pathogens in hospitals and implantable devices: Gram-negative *E. coli* and Gram-positive *S. aureus* bacteria, using the live/dead staining method [47]. Viability was estimated as the ratio of viable bacteria to the total number of bacteria attached to the sample surface, and the results are shown in Figure 8 [47]. About 80% of *S. aureus* were alive on the control, whilst the viability on the neat Ge prepared via plasma polymerization with radio frequency powers of 10 and 50 W were 53% and 50%, and those of the corresponding nanocomposites with ZnO were 31% and 42%, respectively (Figure 8a). Likewise, 81% of *E. coli* were viable on the control surface, whereas the viability on the neat oil were 60% and 76%, and for the composites were 33% and 44%, respectively.

The antibacterial action of VO-based polymers is related to their surface chemistry and nanoscale topography. The functional groups of the VO (ie. hydroxyl, carboxylic, methyl) are reported to disturb the microbial growth and avoid a good anchoring of microbial cells [51]. However, pristine VO showed low antibacterial activity versus both Gram-positive and Gram-negative bacteria. A plausible explanation could be that plasma treatment would have worsened the antimicrobial activity of the neat oil due to the crosslinking of many molecules, making them incapable of interacting with the cell membranes [52].

In contrast, the Ge-based ZnO nanocomposites revealed enhanced bactericidal performance, corroborating that ZnO NPs were straightforwardly implicated in the inhibition of the pathogens. The antimicrobial mechanisms of MO-NPs, as described earlier, differ from those of the pristine polymers. Despite both being capable of anchoring to the outer microorganism surface—thus modifying the permeability of cell walls—NPs, due to their extremely small size, are also able to enter the cell, thus gathering in the cytoplasm, disrupting cellular activities and provoking membrane disruption. Besides, upon interaction with the bacteria cells, ZnO NPs can produce ROS, as detailed above; which can deteriorate the proteins, peptidoglycan, ribosomes, and DNA of the bacteria, causing inhibition of enzymatic activities and amino acid production, thus leading to cell lysis. This accounts for the improved performance observed for the nanocomposites compared to the neat Ge. In addition, it is possible the existence of synergistic effects of both composite components on inhibiting bacteria viability, as reported for nanocomposites reinforced with graphene and Ag NPs [53] or ZnO and Ag NPs [54], which were more efficient versus pathogens than the individual components alone.

On the other hand, the bactericide action of Zn/Ge 10 W was greater compared to Zn/Ge 50 W, probably related to the different NP size, since the smaller power during the plasma polymerization led to smaller particles, as discussed in Section 3.4, which are more likely to penetrate the bacteria cell. Furthermore, the lower resistance of *S. aureus* against ZnO NPs compared to *E. coli* is consistent with previous reports [55], ascribed to the outer membrane layer external to the peptidoglycan cell wall in the Gram-negative bacteria that can provide better resistance to ZnO penetration. Nonetheless, there is no agreement in the literature on this point, since ZnO NPs are synthesized with different dimensions, morphologies, surface modifications, surface defects, etc., that strongly condition their antibacterial action.

**Figure 9:**
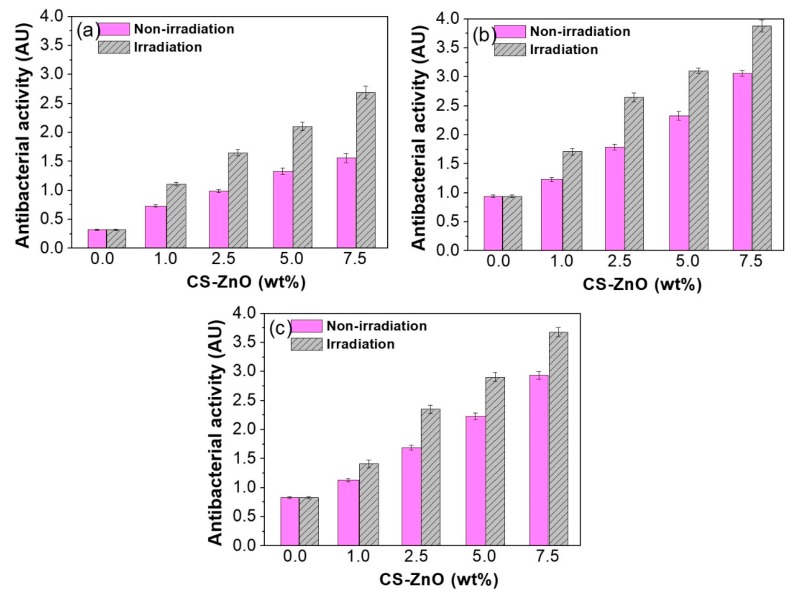
Antibacterial activity of CO/CS-ZnO nanocomposites versus (**a**) *E. coli*, (**b**) *S. aureus*, and (**c**) *M. luteus*. Solid and dashed bars correspond to experiments performed without and with UV light irradiation, respectively. Reprinted from [18], copyright 2015, with permission from the American Chemical Society.

CO/CS-ZnO nanocomposites were tested against Gram-positive *S. aureus* and *M. luteus* and Gram-negative *E. coli* with and without UV illumination (Figure 9) [18]. Experiments were performed following the ISO 22196:2007 standard. Samples were sterilized in an autoclave and then submerged in a nutrient broth of ~2.0×106 colony forming units (CFU)/mL. After incubation at 37 ºC for 24 h, the number of CFU per sample was calculated manually. The antibacterial activity was estimated as: log(viable cell count in the control/viable cell count in the composite), where the control was a flask containing bacteria and no sample. It has been stipulated that efficient antibacterial activity should be higher than 2.

Cured CO shows some antimicrobial action versus the three bacteria tested, being the activity stronger against Gram-positive ones both with and without UV light. A number of works have confirmed that fatty acids can restrain the bacterial growth [56], and even their mechanism is not well understood up to date, it appears to be connected with the electron transport disruption and oxidative phosphorylation at the cell membrane. Moreover, their bactericide action could arise from enzymatic activity inhibition and nutrient uptake or lysis of bacterial cells. More importantly, the antimicrobial activity increases as the CS-ZnO loading rises under both conditions tested. Nonetheless, while *E. coli* activity was restrained for NP contents ≥ 5.0 wt % under UV irradiation, *S. aureus* and *M. luteus* were inhibited by all the nanocomposites except for that with 1.0 wt % loading. This is consistent with the results previously discussed for Ge/Zn nanocomposites [47], corroborating that the antibacterial action is strongly influenced by the structural and chemical composition of the bacterial cell. Interestingly, CO/ZnO composites also show antibacterial activity without UV light, being only slightly toxic to *E. coli* while more effective against *S. aureus* and *M. luteus* (Figure 9b,c). Despite the generation of ROS is supposed to be the key reason for the antibacterial activity of ZnO NPs in the presence of UV light, the cause of their bactericidal effect without irradiation is still unknown; it is not likely related to ROS production albeit dependent on the ZnO attachment to bacterial cell walls, and increasing concentrations of Zn^2^⁺ ions in the bacterial cytoplasm due to local dissolution of the attached ZnO.

Noticeably, the biocide action found for these composites is significantly higher than that found for Ge/Zn nanocomposites prepared via plasma polymerization [47], indicating that both the functionalization of the nanoparticles and the nanocomposite synthesis process can play a key role in the biocide action. In particular, the biological activity of ZnO has been reported to be strongly dependent on the surface functional groups. Thus, Betancourt-Galindo et al. [57] modified the ZnO NPs by di-functional alcohol, and the resulting NPs showed improved performance, and were used in medical devices. In addition, it should be highlighted the exceptional antimicrobial properties of CS [58]. This polysaccharide is successful against both Gram-negative and Gram-positive microorganisms, while its efficiency is conditioned by factors such as its molecular weight, degree of deacetylation, and concentration. It is supposed to be able to go through the bacterial cell wall by means of pervasion and fabrication of a polymer membrane on the cell wall surface. Furthermore, its positively charged amino groups are prone to interact with the negatively charged bacterial membranes, resulting in protein leakage and affecting the phospholipid bilayer structure, thereby altering its permeability [59]. CS hybrid materials incorporating metal and MO-NPs have been prepared with outstanding properties derived from synergistic effects [60,61,62,63,64]. It has been reported that CS or modified CS combined with Ag leads to hydrogels with better antimicrobial activity [63]. Besides, mixtures of CS, polyvinylpyrrolidone and nanosized TiO_2_ [61] or Ag_2_O [62] exhibit superb wound healing and antimicrobial characteristics. ZnO–CS complexes with antimicrobial and antibiofilm properties have been also prepared by nano spray drying and precipitation methods [64].

**Figure 10:**
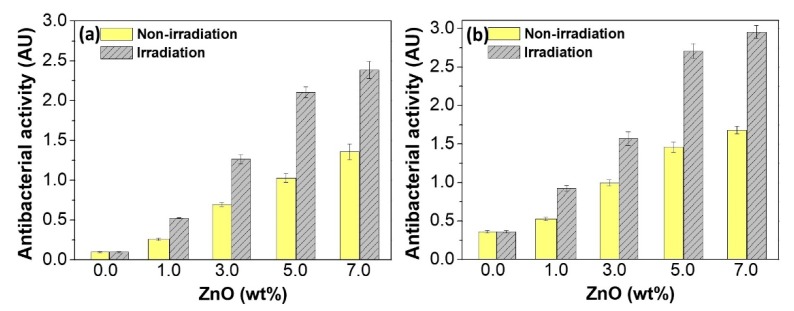
Antibacterial activity of ESO/ZnO nanocomposites against (**a**) *E. coli* and (**b**) *S. aureus*. Solid and dashed bars as indicated in Figure 9. Reprinted from [17], copyright 2014, with permission from the American Chemical Society.

The antibacterial activity of ESO/ZnO nanocomposites was also tested against *E. coli* and *S. aureus* with and without UV light (Figure 10) [17], following the same protocol as indicated earlier [18]. Neat ESO exhibits a little antimicrobial effect, ~0.1 and 0.4 versus *E. coli* and *S. aureus* respectively, under both conditions examined, the effect being smaller than that found for CO [18]. The differences are likely related to their chemical structure, since CO comprises a large number of reactive hydroxyl groups that improve the antibacterial activity. This is consistent with a previous study on ESO modified with quaternary ammonium salts and hydroxyl groups onto the backbone [65], which was reacted with different diisocyanate monomers to prepare polyurethane coatings that showed considerably enhanced antibacterial activity (about 95% bacterial reduction). The positive charges of the quaternary ammonium salts can interact with negatively charged surfaces of both Gram-positive and Gram-negative bacteria via electrostatic forces, which promote NP penetration Thus, the antimicrobial activity of VOs is confirmed to be strongly dependent on their surface functional groups, as mentioned earlier. 

On the other hand, as found for the other nanocomposites reinforced with ZnO NPs [18,47], the antibacterial action increases upon rising NP loading both in the presence and the absence of UV light. This trend arises from the uniform nanoparticle dispersion within the VO matrix. Thus, the higher the NP content, the higher the effective surface area available for interaction with the bacteria cell. Again, a more efficient bactericide action is found versus Gram-positive bacteria, related to the differences in structure and chemical composition of the cell walls. The thicker and multipart structure of *E. coli* should minimize the harm from oxidation radicals. It should be noted that for both bacteria tested, the antibacterial action of ESO/ZnO nanocomposites is weaker than that found for CO/CS-ZnO ones [18], likely due to the functionalization of the NPs, as discussed earlier and the synergistic effect of CS and ZnO. Improved bactericide action has also been reported upon functionalization of other nanomaterials like graphene oxide [66] or boron nitride nanotubes [67] with biocompatible polymers such as polyethylene glycol.

### 4.2. Antimicrobial Effect of Titanium Oxide-Reinforced Nanocomposites

**Figure 11:**
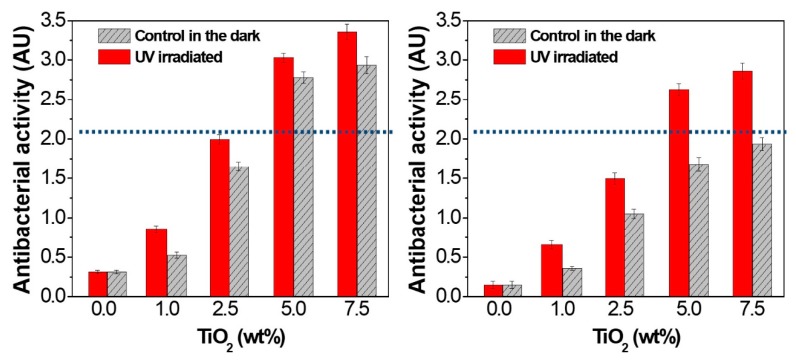
Antibacterial action of AELO/TiO_2_ nanocomposites versus *S. aureus* (left) and *E. coli* (right). The dotted line shows the limit for effective antibacterial action. Reprinted from [46], copyright 2015, with permission from the Royal Society of Chemistry.

Following the same protocol described above, the antibacterial action of AELO/ZnO nanocomposites was also investigated versus *S. aureus* and *E. coli* under both UV light and dark conditions (Figure 11) [46]. For both bacteria, AELO only exerts a minor antimicrobial action, about 0.15 and 0.3 respectively, the effect being alike under both conditions, and slight smaller than that found for ESO [17] and CO-based [18] nanocomposites. This is in agreement with studies on the bactericidal activity of VOs, which found a strong dependence on their content of phenolic compounds, together with their unsaturation degree and chain length: the longer the chain, the stronger the bacterial action is [68]. 

As observed for nanocomposites reinforced with ZnO NPs, the antibacterial action rises as the NP loading rises, both with and without UV light. It is expected that higher TiO_2_ loadings will lead to more NPs in contact with the bacteria, and in consequence, produce more toxicity. Furthermore, the state of NP dispersion also conditions the bactericide action: the more homogenous the NP dispersion, the better the antibacterial property. Thus, the nanocomposite with 7.5 wt % TiO_2_ presents similar action to that with 5.0 wt % due to the presence of small clusters as corroborated by SEM (Figure 2). Again, stronger bactericide effect is found against Gram-positive bacteria, as also reported for TiO_2_/poly(lactic-co-glycolic acid) PLGA composites [69]. The antibacterial effect could be attributed to inactivation of cellular enzyme and DNA by the TiO_2_ NPs causing small pores in the bacterial cell wall that result in increased permeability and cell death. Furthermore, oxidative damage provoked by ROS produced via redox reactions between adsorbed species (i.e., water, oxygen) and electrons and holes generated on irradiation of TiO_2_ with UV light have also occurred. Reaction of the holes in the valence band with H_2_O or hydroxide ions on the surface results in the formation of •OH radicals, whilst electrons in the conduction band react with O= to yield superoxide ions •O^2−^. Both hydroxyl and superoxide ions can react with the phospholipid components of the bacteria cell membrane, thereby causing the deterioration of the membrane and finally cell lyses and bacteria inactivation [70]. 

Experimental data confirm that TiO_2_-reinforced composites possess antibacterial activity under dark conditions, pointing towards other mechanism of bacterial inactivation besides the photocatalytic process. These could include NP-bacteria surface physicochemical interactions that injure the cells; redox reactions at the NP–cell interface concerning reduction of Ti(IV) to Ti(III) that provoke oxidative degradation of the cell membrane; internalization of the NPs in the cell and bacteria damage from the interior; bacterial phagocytosis by the NPs resulting in cell death [71]. Although the precise mechanism is not known up to date, the bacteria-TiO_2_ contact appears to be critical for attaining strong bactericide effect.

### 4.3. Antimicrobial Effect of Copper Oxide-Reinforced Nanocomposites

**Figure 12:**
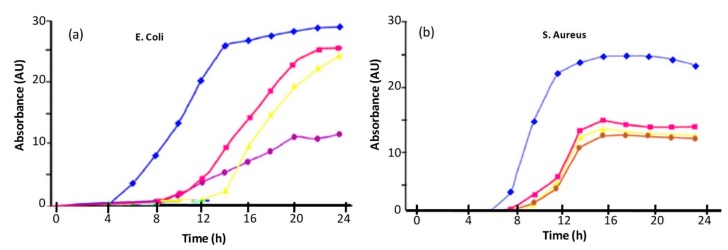
Absorbance versus time showing the effect of LO/CuO nanocomposites on the growth of *E. coli* (a) and *S. aureus* (b). **–** control; – 0.04 % CuO; – 0.05 % CuO; – and –0.06 % CuO; Adapted from [50], copyright 2013, with permission from Elsevier.

To determine the bactericide effect of LO/CuO nanocomposites, *E. coli* and *S. aureus* bacteria were cultured for 24 h at 37 °C. At certain times aliquots were taken and growth was followed turbidometrically at 595 nm with a spectrophotometer [50]. Results are shown in Figure 12. 

The growth curves indicated three regions: initial phase, active lag phase and stationary phase. The absorbance for the control (only bacteria) revealed that *E. coli* and *S. aureus* attained the stationary phase after 14 and 12 h, respectively. A similar trend is found for the nanocomposites, although the lag phase was extended by an average of 6 h in the case of *E. coli*, while for *S. aureus* it remained almost the same. More importantly, in the case of *E. coli*, about 17, 24, and 62% growth was inhibited compared to the control for nanocomposites with 0.04, 0.05, and 0.06% CuO, respectively, showing and increase in bactericide action with increasing NP loading. In contrast, for *S. aureus*, the growth inhibition was in the range of 60–55% for the three nanocomposites.

The antibacterial action of LO derived polyol-CuO nanocomposites seems to arise from the disruption of membrane integrity [72]. Electrostatic interactions can initially take place between positive residue in the composites and negative charges onto the bacteria surface, provoking the adhesion of the nanocomposites to the surface. Then, the polymeric chains could phagocytize the bacteria, and simultaneously the CuO would bind to the lipid and peptidoglycan layers, causing the cell death. Thus, as the NP content increased, the bactericide action improved, though in the case of *S. aureus* the improvement with increasing loading was slightly significant, likely because a high efficiency was already attained for the lowest NP content. Furthermore, this behavior could be the result of their tendency to form clusters like bunches of grapes, which impedes the whole penetration of the nanocomposites to the core of the cluster [50]. In addition, ROS formation can take place by released Cu^2+^; in the presence of either superoxide or other reducing agents such as ascorbic acid, Cu^2+^ can be reduced to Cu^+^ catalyzing the formation of •OH radicals from H_2_O_2_ via the Haber–Weiss reactions [41]. •OH is the most powerful oxidizing radical reacting with almost every biomolecule. It can start oxidative damage by abstracting the hydrogen both from an amino-bearing carbon to form a carbon centered protein radical and from an unsaturated fatty acid to form a lipid radical. Moreover, it is expected that in addition to the effect of CuO, the hydrophobic nature of LO and its fatty acid chains also boost the bactericide action.

### 4.4. Antimicrobial Effect of Iron Oxide-Reinforced Nanocomposites

**Figure 13:**
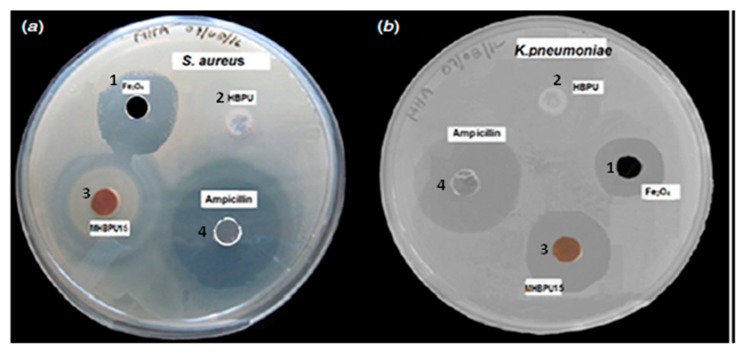
Inhibition zone showing the antibacterial activity against of *S. aureus* (**a**) and *K. pneumoniae* (**b**), respectively, of Fe_3_O_4_ (**1**), sunflower oil derived-polyurethane (**2**), the corresponding nanocomposite with 15 wt % loading (**3**), and the control (**4**). Adapted from [48], copyright 2013, with permission from IOP publishing.

The antibacterial action of sunflower oil-derived HBPU/Fe_3_O_4_ nanocomposites was tested against *S. aureus* and *K. pneumonia* bacteria via the agar well diffusion method [73]. The microorganisms were incubated for 24 h at 37 °C. Upon incubation, the inhibition zone for the different samples was calculated, using ampicillin as control, and results are shown in Figure 13 [48].

The neat polyurethane derived from the VO did not have antibacterial activity while Fe_3_O_4_ NPs alone showed inhibition zones of 13 and 11 mm versus Gram-positive *S. aureus* and Gram-negative *K. pneumoniae*, respectively. Surprisingly, the nanocomposite with 15 wt % NP displayed a zone of inhibition of 15 and 13 mm for the indicated bacteria. This corresponds to normalized widths of the antimicrobial “halo” (*nw_halo_*) of 0.43 and 0.42, respectively, calculated from the diameter of the inhibition zone (*d_iz_*) and the disk diameter (*d*) as: [(*d_iz_* – d)/2]/d [74]. These results indicate that the NPs provided bactericide action to the VO-based polymer, and this activity increased with the rise in NP loading, as reported for other nanocomposites based on VOs discussed earlier. Nonetheless, the bactericide action found for this nanocomposite seems smaller compared to those based on semiconducting MO-NPs: ZnO [17,18,47], TiO_2_ [46], or CuO [50], which could be related to the fact that in the semiconductors electron–hole pairs are formed, and the holes can split water molecules and cause ROS formation. In the case of magnetite NPs, the mechanism of bactericide action should be different (i.e., via Fenton-like and Haber–Weiss reactions, as discussed earlier), and albeit ROS could also be generated (mainly H_2_O_2_ when Fe^2+^ responded to oxygen that interacts with the outer bilayer of bacteria, thus entering the cell membrane and causing bacterial disruption), these would be less harmful or would be produced in a less efficient way. Furthermore, combined mechanism can also take place. Thus, the ferrous irons can subsequently react with the produced H_2_O_2_ through Fenton reaction, thus leading to hydroxyl radicals that damage the bacteria cell wall. 

**Figure 14:**
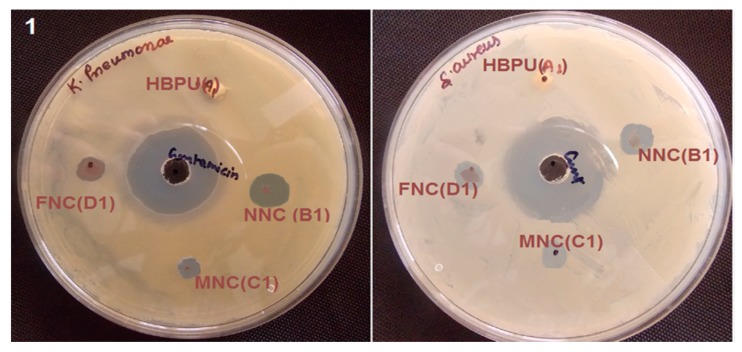
Inhibition zone showing the antibacterial activity against *K. pneumonia* and *S. aureus* for the neat polyurethane derived from the oil (HBPU), NNC, FNC, and MNC. The nomenclature is indicated in Figure 7. Reprinted from [49], copyright 2014, with permission from the Royal Society of Chemistry.

In contrast, a higher bactericide action was found for similar nanocomposites reinforced with Fe_3_O_4_–MWCNT nanohybrids (NNC in Figure 14 [49]), which showed an inhibition zone of 22 mm against *K. pneumoniae*, and close to 20 mm for *S. aureus* (*nw_halo_* values of 0.39 and 0.4, respectively), likely arising from the synergistic effect of the NPs and the MWCNTs on the biocide effect. The antibacterial activity of MWCNTs has been ascribed to several mechanisms [75]: disruption of the membrane integrity by strong electrostatic forces between bacterial outer surface and MWCNTs, leading to oxidation of the membrane; ROS generation that induces destruction of the bacterial plasmid DNA; impurity components (i.e., metallic nanoparticles, catalysts, suspension) that are introduced into the MWCNT structure during the synthesis process; bactericidal oxidative stress. Furthermore, the toxicity of MWCNTs is highly influenced by several factors such as diameter, length, residual catalysts, electronic structure, surface functional groups, and surface chemistry [76]. In particular, the tube length is critical for the interactions with the cell membrane: the shorter the tube, the more chances there are for interaction between open ends of the nanotubes and the bacteria, leading to extra cell membrane damage, thus the bactericidal performance is stronger. The tube diameter also plays a key role in the bacterial inactivation process. Smaller diameters can damage the cell membrane via cell-surface interactions, while larger diameters (~15–30 nm) mostly interact with bacteria by their side walls [77]. 

In the study by Das et al. [49], the MWCNTs were relatively small and short (~10 nm diameter, length < 5 μm, Figure 7d,e), which could account for their improved antibacterial activity. Furthermore, due to the MWCNT shape, they have less bactericidal action toward rod-shaped bacteria (*K. pneumonia)* when compared to spherical ones (*S. aureus*). Overall, the nanohybrids showed significantly improved performance compared to the nanocomposites reinforced only with Fe_3_O_4_ or MWCNTs.

The antibacterial activities of VO/MO-NPs nanocomposites are summarized in Table 3. Systematically, stronger bactericide effect is found against Gram-positive spherical bacteria compared to Gram-negative rod-shaped ones, ascribed to the structural and chemical compositional differences of their cell walls. For similar NP loadings, stronger antibacterial activity is obtained for smaller NPs, which are more prone to penetrate into the bacteria cell. Nonetheless, the two most important parameters determining the bactericide action appear to be the NP concentration and its level of surface functionalization. Systematically, the higher the NP loading, the more intense the NP-bacteria interactions are, hence the stronger the bactericide effect is. However, in some cases a stabilization of the antibacterial activity is envisaged for NP loadings >7.5 wt%, since NP agglomeration can occur, thus reducing the NP specific surface area, which results in lower NP-bacterial interfacial contact area. NP surface functionalization, in particular with positively charged groups, improves the antibacterial efficiency, since cationic groups are susceptible to interact with the negatively charged bacterial membranes via electrostatic forces, resulting in protein leakage and damaging the phospholipid bilayer structure. Besides, synergistic effects typically take place between the MO-NPs and their surface functional moieties on improving antibacterial activity. Regarding the NP shape, slight influence is predicted, although most of the studies were carried out with spherical or quasi-spherical NPs, hence it is difficult to reach a clear conclusion on this issue. The composition of the VO matrix also influences the antibacterial activity of the nanocomposites: the higher its phenolic content, unsaturation degree, and chain length, the stronger the bacterial action is. Thus, composites based on CO, that comprises 89% of unsaturated ricinoleic acid (Table 2), display the highest antibacterial effectiveness. In contrast, those based on sunflower or geranium oil, with lower level of unsaturation, exert less bactericide action. This is consistent with the fact that neat CO showed bactericide action towards both Gram-positive and Gram-negative bacteria, while neat Ge or the HBPU derived from sunflower oil hardly showed bactericide action. Finally, the nanocomposite synthesis process can also play a key role in the biocide action. Thus, plasma polymerization methods can worsen the antimicrobial activity due to the high level of crosslinking between the molecules, which makes their interaction with the cell membranes more difficult. 

## 5. Conclusions

The incorporation of MO-NPs into VO-based thermosetting polymers is a versatile path to take advantage of their outstanding antimicrobial properties, leading to new bactericide nanocomposite materials with a wider range of applications. The antibacterial properties depend on a number of parameters, including the NP size, shape, level of functionalization and in particular, their concentration in the nanocomposite. It has been shown that the biocide action systematically increases with increasing NP loading, due to larger NP-bacteria interfacial contact area. Moreover, surface functionalization of the NPs or hybridization with other nanomaterials significantly improves their efficiency, in particular when this is carried out with substances that also exhibit antimicrobial activity (i.e., polymers like chitosan or MWCNTs). The main mechanism of action is likely the generation of ROS, which can disrupt cell membranes, damage DNA synthesis, and perturb metabolic paths, finally resulting in cell death. Furthermore, these NPs also exhibit biocide action under dark conditions, corroborating the existence of other modes of action like redox reactions at the NP–cell interface, bacterial phagocytosis, lipid peroxidation, etc. Although several examples related with MO-NPs embedded into VO-derived thermoset polymers have been reported, as discussed throughout this manuscript, additional research is required to support the progress of novel nanocomposites to be used in hospital equipment, in prostheses, or as antibacterial materials in public places. In this regard, novel synthesis approaches based either on commercial matrices or on VOs/MO-NP coatings that could be straightforwardly implemented at an industrial scale should be developed. Future research areas might include scaling up processes, optimization of the NP dispersion, use of novel MO-NPs with antimicrobial properties, different polymeric matrices derived from VOs and toxicity studies. Overall, more research and investment are needed to attain fully sustainable materials with antimicrobial activity as effective substitutes for the existing ones.

## Figures and Tables

**Table 1:** Formulas and structures of the most common fatty acids in vegetable oils

Fatty Acid	Formula	Structure
Palmitic	C_16_H_32_O_2_	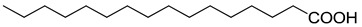
Palmitoleic	C_16_H_30_O_2_	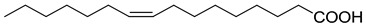
Stearic	C_18_H_36_O_2_	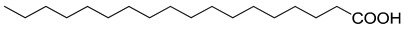
Oleic	C_18_H_34_O_2_	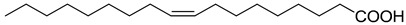
Linoleic	C_18_H_32_O_2_	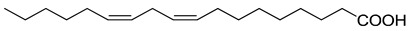
Linolenic	C_18_H_30_O_2_	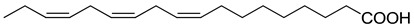
α-Eleostearic	C_18_H_30_O_2_	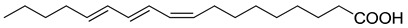

**Table 2:** Properties and fatty acid compositions of the most frequent vegetable oils.

Vegetable Oil	Double Bonds ^a^	Iodine Value ^b^ (mg/100g)		Fatty Acids (%)			
			Palmitic	Stearic	Oleic	Linoleic	Linolenic
Palm	1.7	44–58	42.8	4.2	40.5	10.1	-
Olive	2.8	75–94	13.7	2.5	71.1	10.0	0.6
Groundnut	3.4	80–106	11.4	2.4	48.3	31.9	-
Rapeseed	3.8	94–120	4.0	2.0	56.0	26.0	10
Sesame	3.9	103–116	9.0	6.0	41.0	43.0	1.0
Cottonseed	3.9	90–119	21.6	2.6	18.6	54.4	0.7
Corn	4.5	102–130	10.9	2.0	25.4	59.6	1.2
Soybean	4.6	117–143	11.0	4.0	23.4	53.3	7.8
Sunflower	4.7	110–143	5.2	2.7	37.2	53.8	1.0
Castor ^c^	4.8	83–88	1.3	1.2	4.0	5.2	0.3
Linseed	6.6	168–204	5.5	3.5	19.1	15.3	56

^a^ Average number of double bonds per triglyceride. ^b^ The amount of iodine (mg) that reacts with the double bonds in 100 g of vegetable oil. ^c^ Contains 89% of unsaturated ricinoleic acid

**Table 3:** Antibacterial activity of VO/MO-NPs nanocomposites.

Nanocomposite Type (Processing)	Antibacterial Activity (AU)	Bacteria Strain	Average NP Size (nm)	NP Shape	NP Concentration (wt %)	REF
Zn/Ge 10 (PP)	31 ^a^	*S. aureus*	60	Ball-like	0.79	47
Zn/Ge 10 (PP)	33 ^a^	*E. coli*	60	Ball-like	0.79	47
Zn/Ge 50 (PP)	42 ^a^	*S. aureus*	80	Ball-like	1.57	47
Zn/Ge 50 (PP)	44 ^a^	*E. coli*	80	Ball-like	1.57	47
ESO/ZnO (SM + C)	0.30	*E. coli*	65	Spherical	1.00	17
ESO/ZnO (SM + C)	0.55	*S. aureus*	65	Spherical	1.00	17
ESO/ZnO (SM + C)	0.60	*E. coli*	73	Spherical	3.00	17
ESO/ZnO (SM + C)	0.98	*S. aureus*	73	Spherical	3.00	17
ESO/ZnO (SM + C)	1.12	*E. coli*	80	Spherical	5.00	17
ESO/ZnO (SM + C)	1.47	*S. aureus*	80	Spherical	5.00	17
ESO/ZnO (SM + C)	1.32	*E. coli*	92	Spherical	7.00	17
ESO/ZnO (SM + C)	1.68	*S. aureus*	92	Spherical	7.00	17
AELO/TiO_2_ (IP + C)	0.41	*E. coli*	40	Spherical	1.00	46
AELO/TiO_2_ (IP + C)	0.60	*S. aureus*	40	Spherical	1.00	46
AELO/TiO_2_ (IP + C)	1.12	*E. coli*	42	Spherical	2.50	46
AELO/TiO_2_ (IP + C)	1.62	*S. aureus*	42	Spherical	2.50	46
AELO/TiO_2_ (IP + C)	1.73	*E. coli*	46	Spherical	5.00	46
AELO/TiO_2_ (IP + C)	2.68	*S. aureus*	46	Spherical	5.00	46
AELO/TiO_2_ (IP + C)	1.92	*E. coli*	64	Spherical	7.50	46
AELO/TiO_2_ (IP + C)	2.81	*S. aureus*	64	Spherical	7.50	46
CO/CS-ZnO (SM + C)	0.75	*E. coli*	168	Quasi-spherical	1.00	18
CO/CS-ZnO (SM + C)	1.20	*S. aureus*	168	Quasi-spherical	1.00	18
CO/CS-ZnO (SM + C)	1.10	*M. luteus*	168	Quasi-spherical	1.00	18
CO/CS-ZnO (SM + C)	0.98	*E. coli*	177	Quasi-spherical	2.50	18
CO/CS-ZnO (SM + C)	1.80	*S. aureus*	177	Quasi-spherical	2.50	18
CO/CS-ZnO (SM + C)	1.71	*M. luteus*	177	Quasi-spherical	2.50	18
CO/CS-ZnO (SM + C)	1.32	*E. coli*	175	Quasi-spherical	5.00	18
CO/CS-ZnO (SM + C)	2.30	*S. aureus*	175	Quasi-spherical	5.00	18
CO/CS-ZnO (SM + C)	2.24	*M. luteus*	175	Quasi-spherical	5.00	18
CO/CS-ZnO (SM + C)	1.55	*E. coli*	180	Quasi-spherical	7.50	18
CO/CS-ZnO (SM + C)	3.05	*S. aureus*	180	Quasi-spherical	7.50	18
CO/CS-ZnO (SM + C)	2.98	*M. luteus*	180	Quasi-spherical	7.50	18
LO/CuO (SL)	17 ^b^	*E. coli*	50	Quasi-spherical	0.04 ^c^	50
LO/CuO (SL)	55 ^b^	*S. aureus*	50	Quasi-spherical	0.04 ^c^	50
LO/CuO (SL)	24 ^b^	*E. coli*	56	Quasi-spherical	0.05 ^c^	50
LO/CuO (SL)	57^b^	*S. aureus*	56	Quasi-spherical	0.05 ^c^	50
LO/CuO (SL)	62 ^b^	*E. coli*	59	Quasi-spherical	0.06 ^c^	50
LO/CuO (SL)	60 ^b^	*S. aureus*	59	Quasi-spherical	0.06 ^c^	50
HBPU/Fe_3_O_4_ (SC + C)	0.43^d^	*S. aureus*	9	Spherical	15.0	48
HBPU/Fe_3_O_4_ (SC + C)	0.42 ^d^	*K. pneu-moniae*	9	Spherical	15.0	48
HBPU/Fe_3_O_4_-CNT (SC + C)	0.40 ^d^	*S. aureus*	11	Spherical	15.0	49
HBPU/Fe_3_O_4_-CNT (SC + C)	0.39 ^d^	*K. pneu-moniae*	11	Spherical	15.0	49

^a^ Percentage of cell viability; ^b^ Percentage of cell growth inhibition; ^c^ Concentration in mol; ^d^ Normalized width of the antimicrobial “halo”; PP: plasma polymerization; SM: solution mixing; IP: in situ polymerization; SC: solution casting; C: curing; SL: solventless one-pot reaction.

## References

[1] Lligadas G., Ronda J.C., Galià M., Cádiz V. (2013). Renewable polymeric materials from vegetable oils: A perspective. Mater. Today.

[2] Babu R.P., O’Connor K., Seeram R. (2013). Current progress on bio-based polymers and their future trends. Prog. Biomater..

[3] Raquez J.-M., Deléglise M., Lacrampe M.-F., Krawczak P. (2010). Thermosetting (bio)materials derived from renewable resources: A critical review. Prog. Polym. Sci..

[4] Gunstone F. (1996). Fatty Acid & Lipid Chemistry.

[5] Belgacem M.N., Gandini A. (2008). Monomers, Polymers and Composites from Renewable Resources.

[6] Allen R.R., Formo M.V., Krishnamurthy R.G., McDermott G.N., Norris F.A., Sonntag N.O.V. (1982). Bailey’s Industrial Oil and Fat Products.

[7] Mosiewicki M.A., Aranguren M.I. (2013). A short review on novel biocomposites based on plant oil precursors. Eur. Polym. J..

[8] Meier M.A.R., Metzger J., Schubert U.S. (2007). Plant oil renewable resources as green alternatives in polymer science. Chem. Soc. Rev..

[9] Wool R.P. (2005). Biobased Polymers and Composites.

[10] Alam M., Akram D., Sharmin E., Zafar F., Ahmad S. (2014). Vegetable oil based eco-friendly coating materials: A review article. Arab. J. Chem..

[11] Xia Y., Zhang Z., Kessler M.R., Brehm-Stecher B., Larock R.C. (2012). Antibacterial Soybean-Oil-Based Cationic Polyurethane Coatings Prepared from Different Amino Polyols. Chem. Sus. Chem..

[12] Garrison T.F., Zhang Z., Kim H., Mitra D., Xia Y., Pfister D.P., Brehm-Stecher B., Larock R.C., Kessler M.R. (2014). Thermo-Mechanical and Antibacterial Properties of Soybean Oil-Based Cationic Polyurethane Coatings: Effects of Amine Ratio and Degree of Crosslinking. Macromol. Mater. Eng..

[13] Liang H., Liu L., Lu J., Chen M., Zhang C. (2018). Castor oil-based cationic waterborne polyurethane dispersions: Storage stability, thermo-physical properties and antibacterial properties. Ind. Crops. Prod..

[14] Wang R., Schuman T.P. (2013). Vegetable oil-derived epoxy monomers and polymer blends: A comparative study with review. Express Polym. Lett..

[15] Zhu L., Wool R.P. (2006). Nanoclay reinforced bio-based elastomers: Synthesis and characterization. Polymer.

[16] Lu Y., Larock R.C. (2006). Novel Biobased Nanocomposites from Soybean Oil and Functionalized Organoclay. Biomacromolecules.

[17] Díez-Pascual A.M., Díez-Vicente A.L. (2014). Epoxidized Soybean Oil/ZnOBiocomposites for Soft Tissue Applications: Preparation and Characterization. ACS Appl. Mater. Interfaces.

[18] Díez-Pascual A.M., Díez-Vicente A.L. (2015). Wound Healing Bionanocomposites Based on Castor Oil Polymeric Films Reinforced with Chitosan-Modified ZnO Nanoparticles. Biomacromolecules.

[19] Deka H., Karak N., Kalita R.D., Buragohain A.K. (2010). Bio-based thermostable, biodegradable and biocompatible hyperbranched polyurethane/Ag nanocomposites with antimicrobial activity. Polym. Degrad. Stab..

[20] Gold K., Slay B., Knackstedt M., Gaharwar A.K. (2018). Antimicrobial Activity of Metal and Metal-Oxide Based Nanoparticles. Adv. Therap..

[21] Seil J.T., Webster T.J. (2012). Antimicrobial applications of nanotechnology: Methods and literature. Int. J. Nanomed..

[22] Brunner T.J., Wick P., Manser P., Spohn P., Grass R.N., Limbach L.K., Bruinink A., Stark W.J. (2006). In Vitro Cytotoxicity of Oxide Nanoparticles:  Comparison to Asbestos, Silica, and the Effect of Particle Solubility. Environ. Sci. Technol..

[23] Díez-Pascual A.M., Xu C., Luque R. (2014). Development and characterization of novel poly(ether ether ketone)/ZnObionanocomposites. J. Mater. Chem. B.

[24] Coleman A., Jagadish C., Jagadish C., Pearton S. (2006). Basic properties and applications of ZnO—Chapter 1. Zinc Oxide Bulk, Thin Films and Nanostructures.

[25] Díez-Pascual A.M., Díez-Vicente A.L. (2014). Development of Nanocomposites Reinforced with CarboxylatedPoly(ether ether ketone) Grafted to Zinc Oxide with Superior Antibacterial Properties. ACS Appl. Mater. Interfaces.

[26] Díez-Pascual A.M., Díez-Vicente A.L. (2014). Poly(3-hydroxybutyrate)/ZnOBionanocomposites with Improved Mechanical, Barrier and Antibacterial Properties. Int. J. Mol. Sci..

[27] Hajipour M.J., Fromm K.M., Akbar Ashkarran A., Jimenez de Aberasturi D., Larramendi I.R.D., Rojo T., Serpooshan V., Parak W.J., Mahmoudi M. (2012). Antibacterial properties of nanoparticles. Trends Biotechnol..

[28] Díez-Pascual A.M., Díez-Vicente A.L. (2014). ZnO-Reinforced Poly(3-hydroxybutyrate-co-3-hydroxyvalerate) Bionanocomposites with Antimicrobial Function for Food Packaging. ACS Appl. Mater. Interfaces.

[29] Zheng K., Setyawati M.I., Leong D.T., Xie J. (2017). Antimicrobial Gold Nanoclusters. ACS Nano.

[30] Padmavathy N., Vijayaraghavan R. (2008). Enhanced bioactivity of ZnO nanoparticles-an antimicrobial study. Sci. Technol. Adv. Mater..

[31] Díez-Pascual A.M., Díez-Vicente A.L. (2014). High-Performance Aminated Poly(phenylene sulfide)/ZnO Nanocomposites for Medical Applications. ACS Appl. Mater. Interfaces.

[32] Díez-Pascual A.M., Díez-Vicente A.L. (2014). Effect of TiO2 nanoparticles on the performance of polyphenylsulfone biomaterial for orthopaedic implants. J. Mater. Chem. B.

[33] Pelaez M., Nolan N.T., Pillai S.C., Seery M.K., Falaras P., Kontos A.G., Dunlop P.S.M., Hamilton J.W.J., Byrne J.A., O’Shea K. (2012). A review on the visible light active titanium dioxide photocatalysts for environmental applications. Appl. Catal. B.

[34] Fadeel B., Garcia-Bennett A.E. (2010). Better safe than sorry: Understanding the toxicological properties of inorganic nanoparticles manufactured for biomedical applications. Adv. Drug Deliv. Rev..

[35] Díez-Pascual A.M., Díez-Vicente A.L. (2015). Nano-TiO2 Reinforced PEEK/PEI Blends as Biomaterials for Load-Bearing Implant Applications. ACS Appl. Mater. Interfaces.

[36] Galib M.B., Mashru M., Jagtap C., Patgiri B.J., Prajapati P.K. (2011). Therapeutic potentials of metals in ancient India: A review through Charaka Samhita. J. Ayurveda Inter. Med..

[37] Allahverdiyev A.M., Kon K.V., Abamor E.S., Bagirova M., Rafailovich M. (2011). Coping with antibiotic resistance: Combining nanoparticles with antibiotics and other antimicrobial agents. Expert Rev. Anti InfecyTher..

[38] Magdolenova Z., Collins A., Kumar A., Dhawan A., Stone V., Dusinska M. (2014). Mechanisms of genotoxicity. A review of in vitro and in vivo studies with engineered nanoparticles. Nanotoxicology.

[39] Richardson H.W. (2000). Copper Compounds. Ullmann’s Encyclopedia of Industrial Chemistry.

[40] Moreno J.L.V., Padama A.A.B., Kasai H. (2014). A density functional theory-based study on the dissociation of NO on a CuO(110) surface. Cryst. Eng. Comm..

[41] Angelé-Martínez C., Nguyen K.V.T., Ameer F.S., Anker J.N., Brumaghim J.L. (2017). Reactive oxygen species generation by copper(II) oxide nanoparticles determined by DNA damage assays and EPR spectroscopy. Nanotoxicology.

[42] Yamamoto K., Kawanishi S. (1989). Hydroxyl free radical is not the main active species in site-specific DNA damage induced by copper (II) ion and hydrogen peroxide. J. Biol. Chem..

[43] Blaney L. (2007). Magnetite (Fe3O4): Properties, Synthesis, and Applications. Lehigh Rev..

[44] Xiaodi L., Zhiguo Z., Yufeng T., Bingyu L. (2013). Review on the Synthesis and Applications of Fe3O4 Nanomaterials. J. Nanomater..

[45] Arakha M., Pal S., Samantarrai D., Panigrahi T.K., Mallick B.C., Pramanik K., Mallick B., Jha S. (2015). Antimicrobial activity of iron oxide nanoparticle upon modulation of nanoparticle-bacteria interface. Sci. Rep..

[46] Díez-Pascual A.M., Díez-Vicente A.L. (2015). Development of linseed oil–TiO2 green nanocomposites as antimicrobial coatings. J. Mater. Chem. B.

[47] Al-Jumaili A., Mulvey P., Kumar A., Prasad K., Bazaka K., Warner J., Jacob M.V. (2019). Eco-friendly nanocomposites derived from geranium oil and zinc oxide in one step approach. Sci. Rep..

[48] Das B., Mandal M., Upadhyay A., Chattopadhyay P., Karak N. (2013). Bio-based hyperbranched polyurethane/Fe3O4nanocomposites: Smart antibacterial biomaterials for biomedical devices and implants. Biomed. Mater..

[49] Das B., Chattopadhyay P., Upadhyay A., Gupta K., Mandal M., Karak N. (2014). Biophysico-chemical interfacial attributes of Fe3O4 decorated MWCNT nanohybrid/bio-based hyperbranched polyurethane nanocomposite: An antibacterial wound healing material with controlled drug release potential. N. J. Chem..

[50] Sharmin E., Zafar F., Akram D., Ahmad S. (2013). Plant oil polyol nanocomposite for antibacterial polyurethane coating. Prog. Org. Coat..

[51] Bazaka K., Jacob M., Truong V.K., Crawford R.J., Ivanova E.P. (2011). The Effect of Polyterpenol Thin Film Surfaces on Bacterial Viability and Adhesion. Polymers.

[52] Bazaka K., Bazaka O., Levchenko I., Xu S., Ivanova E.P., Keidar M., Ostrikov K. (2017). Plasma-potentiated small molecules—Possible alternative to antibiotics?. Nano Futures.

[53] Prasad K., Lekshmi G.S., Ostrikov K., Lussini V., Blinco J., Mohandas M., Vasilev K., Bottle S., Bazaka K., Ostrikov K. (2017). Synergic bactericidal effects of reduced graphene oxide and silver nanoparticles against Gram-positive and Gram-negative bacteria. Sci. Rep..

[54] Sinha R., Karan R., Sinha A., Khare S.K. (2011). Interaction and nanotoxic effect of ZnO and Ag nanoparticles on mesophilic and halophilic bacterial cells. Bioresour. Technol..

[55] Arakha M., Saleem M., Mallick B.C., Jha S. (2015). The effects of interfacial potential on antimicrobial propensity of ZnO nanoparticle. Sci. Rep..

[56] Desbois A., Smith V. (2010). Antibacterial free fatty acids: Activities, mechanisms of action and biotechnological potential. Appl. Microbiol. Biotechnol..

[57] Betancourt-Galindo R., Berlanga Duarte M.L., Puente Urbina B.A., Rodríguez-Fernández O.S., Sánchez-Valdés S. (2010). Surface Modification of ZnO Nanoparticles. Mater. Sci Forum.

[58] Rabea E.I., Badawy M.E., Stevens C.V., Smagghe G., Steurbaut W. (2003). Chitosan as Antimicrobial Agent:  Applications and Mode of Action. Biomacromolecules.

[59] Chung Y., Chen C. (2008). Antibacterial characteristics and activity of acid-soluble chitosan. Bioresour. Technol..

[60] Archana D., Dutta J., Dutta P.K. (2013). Evaluation of chitosan nano dressing for wound healing: Characterization, in vitro and in vivo studies. Int. J. Biol. Macromol..

[61] Archana D., Singh B.K., Dutta J., Dutta P.K. (2013). In vivo evaluation of chitosan–PVP–titanium dioxide nanocomposite as wound dressing material. Carbohyd. Polym..

[62] Archana D., Singh B.K., Dutta J., Dutta P.K. (2015). Chitosan-PVP-nano silver oxide wound dressing: In vitro and in vivo evaluation. Int. J. Biol. Macromol..

[63] Amin K.A.M., Panhuis M.I.H. (2012). Reinforced Materials Based on Chitosan, TiO2 and Ag Composites. Polymers.

[64] Dhillon G., Kaur S., Brar S. (2014). Facile fabrication and characterization of chitosan-based zinc oxide nanoparticles and evaluation of their antimicrobial and antibiofilm activity. Int. Nano Lett..

[65] Bakhshi H., Yeganeh H., Mehdipour-Ataei S., Shokrgozar M., Yari A., Seyyed N. (2013). Synthesis and characterization of antibacterial polyurethane coatings from quaternary ammonium functionalized soybean oil based polyols. Mater. Sci. Eng. C.

[66] Díez-Pascual A.M., Díez-Vicente A.L. (2016). Poly(propylene fumarate)/Polyethylene Glycol-Modified Graphene Oxide Nanocomposites for Tissue Engineering. ACS Appl. Mater. Interfaces.

[67] Díez-Pascual A.M., Díez-Vicente A.L. (2016). PEGylated boron nitride nanotube-reinforced poly(propylene fumarate) nanocomposite biomaterials. RSC Adv..

[68] Muñoz-Bonilla A., Cerrada M.L., Fernandez-Garcia M. (2014). Introduction to Polymeric Antimicrobial Materials. Chapter 1. Polymeric Materials with Antimicrobial Activity: From Synthesis to Applications.

[69] Wu J., Li C., Tsai C., Chou C., Chen D., Wang G. (2014). Synthesis of antibacterial TiO2/PLGA composite biofilms. Nanomed. Nanotechnol..

[70] Cho M., Chung H., Choi W., Yoon J. (2004). Linear correlation between inactivation of E. coli and OH radical concentration in TiO2 photocatalytic disinfection. Water Res..

[71] Nadtochenko V., Denisov N., Sarkisov O., Gumy D., Pulgarin C., Kiwi J. (2006). Laser kinetic spectroscopy of the interfacial charge transfer between membrane cell walls of E. coli and TiO2. J. Photoch. Photobio. A.

[72] Zielecka M., Bujnowska E., Kępska B., Wenda M., Piotrowska M. (2011). Antimicrobial additives for architectural paints and impregnates. Prog. Org. Coat..

[73] Turkoglu A., Duru M.E., Mercan N., Kivrak I., Gezer K. (2007). Antioxidant and antimicrobial activities of Laetiporussulphureus (Bull.) Murrill. Food Chem..

[74] Martí M., Frígols B., Serrano-Aroca A. (2018). Antimicrobial Characterization of Advanced Materials for Bioengineering Applications. J. Vis. Exp..

[75] Pramanik S., Konwarh R., Deka R.C., Aidew L., Barua N., Buragohain A.K., Mohanta D., Karak N. (2013). Microwave-assisted poly (glycidyl methacrylate)-functionalized multiwall carbon nanotubes with a ‘tendrillar’ nanofibrous polyaniline wrapping and their interaction at bio-interface. Carbon.

[76] Jackson P., Jacobsen N.R., Baun A., Birkedal R., Kühnel D., Jensen K.A., Vogel U., Wallin H. (2013). Bioaccumulation and ecotoxicity of carbon nanotubes. Chem. Cent. J..

[77] Arias L.R., Yang L. (2009). Inactivation of bacterial pathogens by carbon nanotubes in suspensions. Langmuir.

